# Supplementary material for: Validation of Sputum Biomarker Immunoassays and Cytokine Expression Profiles in COPD
Source: Biomedicines. 2022 Aug 11;10(8):1949. doi: 10.3390/biomedicines10081949 (PMC9405928; doi:10.3390/biomedicines10081949)
Supplement: Supplementary file 1 [file biomedicines-10-01949-s001.zip › biomedicines-1772404-supplementary.pdf]

Validation of Sputum Biomarker Immunoassays and Cytokine Expression Profiles within COPD Endotypes – *Supplementary Material*

Alex Mulvanny, Caroline Pattwell, Augusta Beech, Thomas Southworth and Dave Singh

Methods:

Sputum Processing Method

Sputum plugs were isolated from saliva and the weight of isolated plugs recorded. Eight volumes of PBS per weight of sputum plugs were added and the sample was vortexed and rocked for 15 minutes. The sample was then centrifuged at 400g for 10 minutes at 4°C, following which 4 volumes of PBS supernatant were removed and stored at -80°C for future cytokine analysis. Four volumes of 0.1% DTT were added, the sample was vortexed and rocked for 15 minutes before being filtered and centrifuged. DTT supernatant was removed and the cell pellet was resuspended in an appropriate volume of PBS prior to cytospin preparation.

Matrix Dilution Method

Matrix dilution was assessed via determination of precision and accuracy of a serial dilution series prepared from individual sputum supernatant samples using the assay specific diluent used to prepare standard calibrators. Samples prepared for MPO analysis were diluted using the kit provided RD5K diluent, and samples analysed using all other assays were prepared using proprietary diluents

In the first attempt, the undiluted sample is used to define the 'nominal' concentration of analyte of interest within that specific sample. Serial dilutions prepared from this sample should demonstrate accuracy within the defined acceptance criteria in order to demonstrate parallelism. If this acceptance criteria are not met, a second attempt is made using the first diluted sample within the serial dilution series to define the nominal concentration.

Matrix Dilution Assessment

Parallelism is assessed via % recovery, which is calculated via comparison of the analyte concentration reported at varying dilutions against the value reported at the proposed MRD.

Analyte concentrations reported following the analysis of samples prepared at dilutions subsequent to the MRD should be within 70-130% of the expected concentration at the relevant dilution

calculated from the concentration reported at the MRD. For example, the expected concentration of a sample prepared at a dilution of 1:2 interpolated from the standard curve should be 50% of that reported following analysis of the same sample undiluted (where the undiluted (neat) sample is acting as the MRD).

Eotaxin data is presented in Supplementary Table 2 and Figure 2 as an example of MRD selection and confirmation from matrix dilution data.

Standard Recovery – Subtraction Method

The subtraction method calculates percentage recovery of spiked recombinant proteins within samples prepared at the assay MRD.

$$Recovery\ (\%) = \left(\frac{Measured\ Concentration - Endogenous\ Concentration}{Spike\ Concentration}\right) x\ 100$$

Limit of Detection (LOD)

Assay sensitivity was determined by running 20 replicates of the appropriate blank calibrator. The raw response was averaged, and this value plus 3 standard deviations was read back from the calibration curve. This concentration defined the assay LOD.

qPCR detection of H.influenzae

Real-time qPCR was performed as previously described [1]. Briefly, DNA was extracted from homogenised sputum samples using QIAamp DNA mini kit (QIAGEN, Crawley, West Sussex, UK); bacterial DNA was stored at -80°C. Real time qPCR was performed on *H. influenzae* (HI)*, M. catarrhalis* (MC)*, S. pneumoniae* (SP) *and P. aeruginosa* (PA) targeting the lipo-oligosaccharide glycosyltransferase-encoding gene (*lgtC*) of *H. influenzae,* the *CopB* outer membrane protein encoding gene of *M. catarrhalis*, the autolysis-encoding gene (*lytA*) of *S. pneumoniae* and the *gyrB* gene of *P. aeruginosa*. The upper limit of HNS colonisation ($3.22x10^5$, $3.72x10^3$, $7.09x10^6$ and $1.68x10^2$ genome copies / mL for HI, MC, SP and PA respectively) was used as a threshold to define bacterial colonisation for individual bacterial species in COPD patients.

The qPCR program consisted of 95°C for 15 minutes, 40 cycles of 95°C for 15 seconds and 60°C for 1 minute with a plate read after every cycle. Fluorescent was detected and captured using an Agilent MX3005P detection system. Automated analysis using ABI.Prism software calculated the Ct values,

Prism Version 9.00 (San Diego, USA), A standard curve of known concentrations of bacteria was used to calculate bacteria level.

HI^{+ve} and HI^{-ve} COPD patients (*n*=15 and 34 respectively) were defined using the upper threshold of the HNS range (3.22 x 10^5 genome copies/mL). Two patients within the HI^{+ve} group were also colonised with MC or SP and a further patient colonised with both MC and SP using the respective individual bacterial species HNS upper threshold.

Results:

Establishment of Calibration Curve

Based on the data collected from three independent standard curve preparations, validated standards and calibration curve regression models were established for each analyte.

The MPO ELISA assay utilised a 10-point standard curve fitted to an unweighted 4-parameter logistic regression model. The IL-8 ELISA assay utilised an 8-point standard curve fitted to an unweighted 4-parameter logistic regression model. The 3-Plex Luminex assay utilised an 11-point standard curve fitted to a weighted 5-parameter logistic regression model. The 27-plex assay utilised standard curves from between 6 and 8 validated standards fitted to a weighted 5-parameter logistic regression model. Analyte specific standards are available in supplementary table 7.

Assay Limits of Quantification and Limits of Detection

Assay LLOQ values for MPO, IL-8 and the 3-Plex Luminex assay were defined using endogenous and spiked sputum supernatants, with 6 replicates analysed alongside validation samples in three independent runs. The average %CV across the runs was <16% (Supplementary Table 8).

Due to the varying endogenous concentrations of analytes within the 27-Plex Luminex assay, it was not possible to use an endogenous sample to determine analyte specific LLOQs. The lowest validated standard calibrator was used to define the assay LLOQ. The average %CV across the panel was <25% (Supplementary Table 8). The reported LLOQ concentration is standard reference material lot number specific for the 27-plex panel.

Assay limits of detection are available in Supplementary Table 7.

Unvalidated Analyte Measurements

Basic FGF, GM-CSF, IL-7, IL-9, IL-12p70 and PDGF-BB were classified as unvalidated measurements due to sub-optimal matrix dilution. VEGF was classified as an unvalidated measurement due to <6 validated standard calibrators. Additionally, PDGF-BB demonstrated <6 validated standard calibrators.

Basic FGF, GM-CSF, IL-7, IL-9 , IL-12p70, PDGF-BB and VEGF data was collected as part of the 27-plex Luminex assay. IL-9 was significantly increased in COPD patients compared to HNS ($p<0.05$, Supplementary Table 13). IL-9 and Basic FGF were significantly correlated with sputum neutrophil percentage in COPD (Supplementary Table 14). Basic FGF was significantly increased in HI^{+ve} COPD compared to HI^{-ve}. IL-9 was significantly increased in HI^{+ve} COPD compared to HNS (Supplementary Figure 4).

Figures:

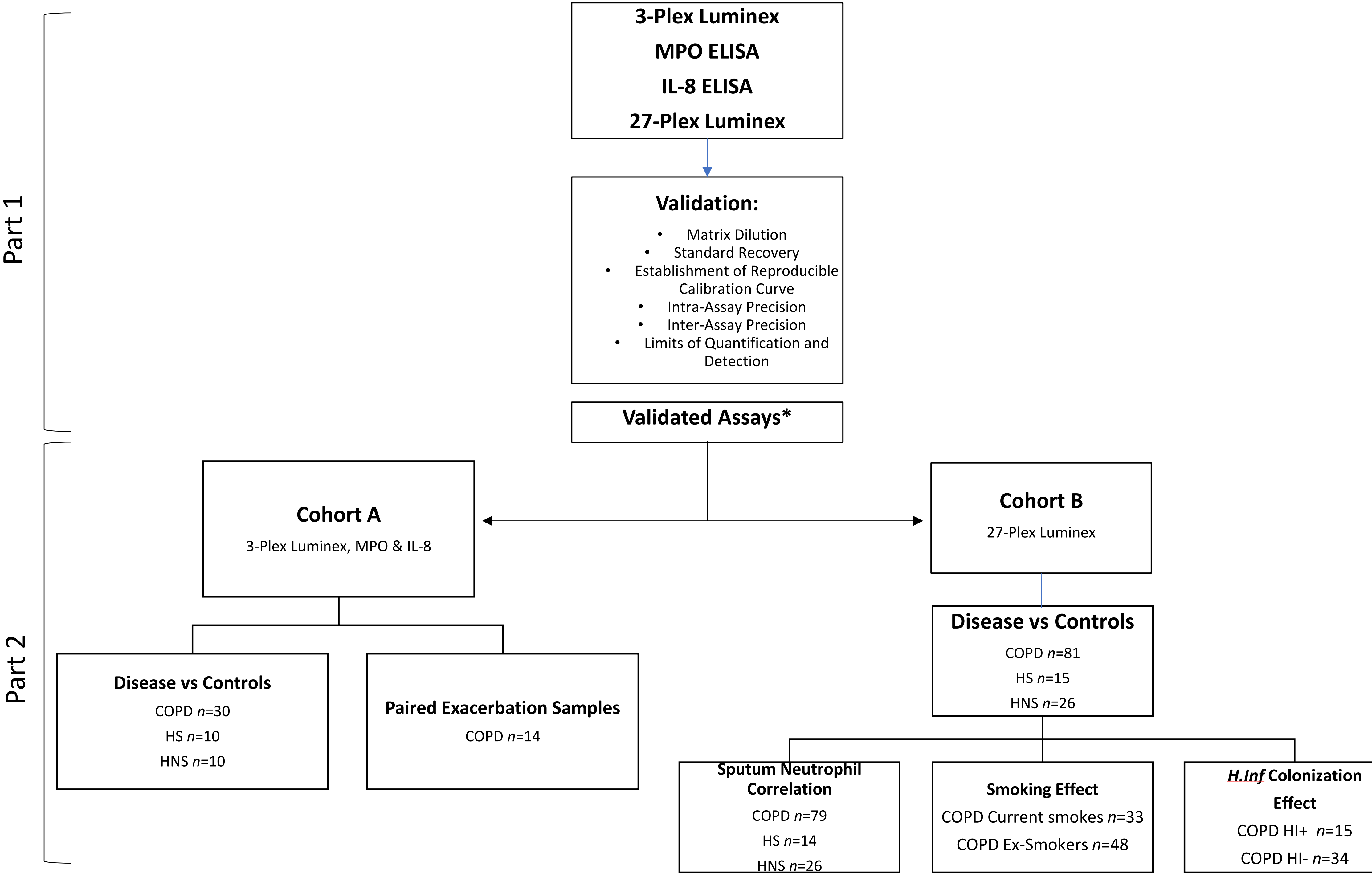

3-Plex Luminex
MPO ELISA
IL-8 ELISA
27-Plex Luminex
Validation:
Matrix Dilution
Standard Recovery
Establishment of Reproducible Calibration Curve
Intra-Assay Precision
Inter-Assay Precision
Limits of Quantification and Detection
Validated Assays*
Part 1
Part 2
Cohort A
3-Plex Luminex, MPO & IL-8
Cohort B
27-Plex Luminex
Disease vs Controls
COPD n=30
HS n=10
HNS n=10
Paired Exacerbation Samples
COPD n=14
Disease vs Controls
COPD n=81
HS n=15
HNS n=26
Sputum Neutrophil Correlation
COPD n=79
HS n=14
HNS n=26
Smoking Effect
COPD Current smokes n=33
COPD Ex-Smokers n=48
H.Inf Colonization Effect
COPD HI+ n=15
COPD HI- n=34

Figure S1: Flow Chart of Study Parts 1 and 2: Flow chart for Part 1: Assay Validation and associated experiments and Part 2: Application of Validated Biomarkers: Cohorts A and B and analysis sub-groups within cohorts.

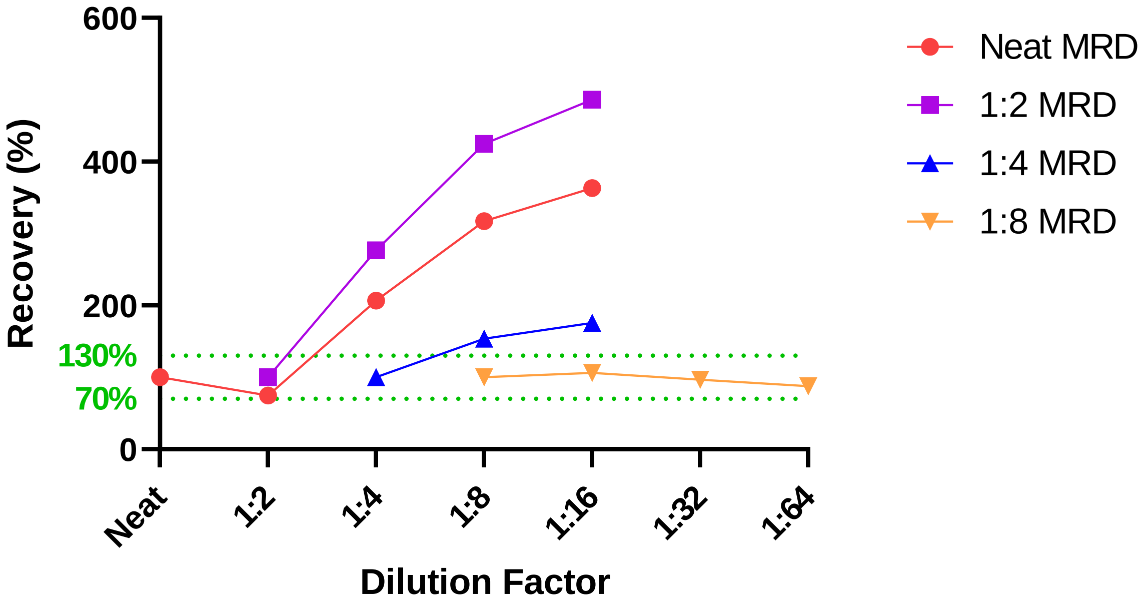

Figure S2: Selection of Assay MRD from Matrix Dilution Assessment: Data is presented as recovery (%) calculated using the concentration reported from samples prepared at dilutions subsequent to the proposed MRD against the concentration expected at the relative dilution using the MRD to calculate the expected concentration.

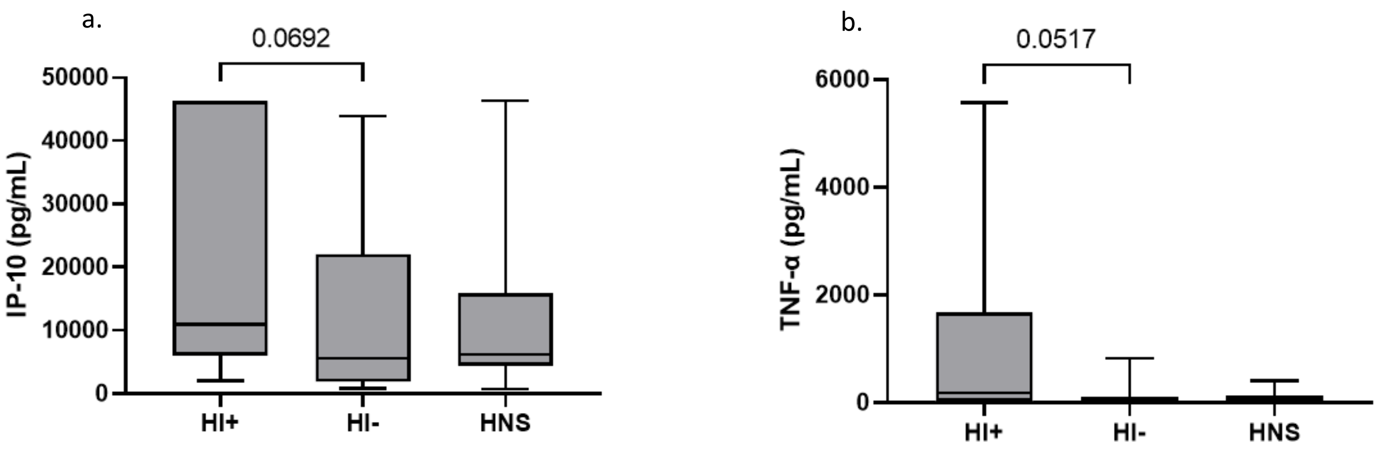

Figure S3: Sputum Biomarkers in COPD HI$^{+ve,}$ COPD HI^{-ve} and HNS: (a) IP-10, data is presented as individual concentrations and the median concentration. (b) TNF-a, data is presented as individual

concentrations and the median concentration, data analysed using Krushkal –Wallis test with Dunns post hoc test.

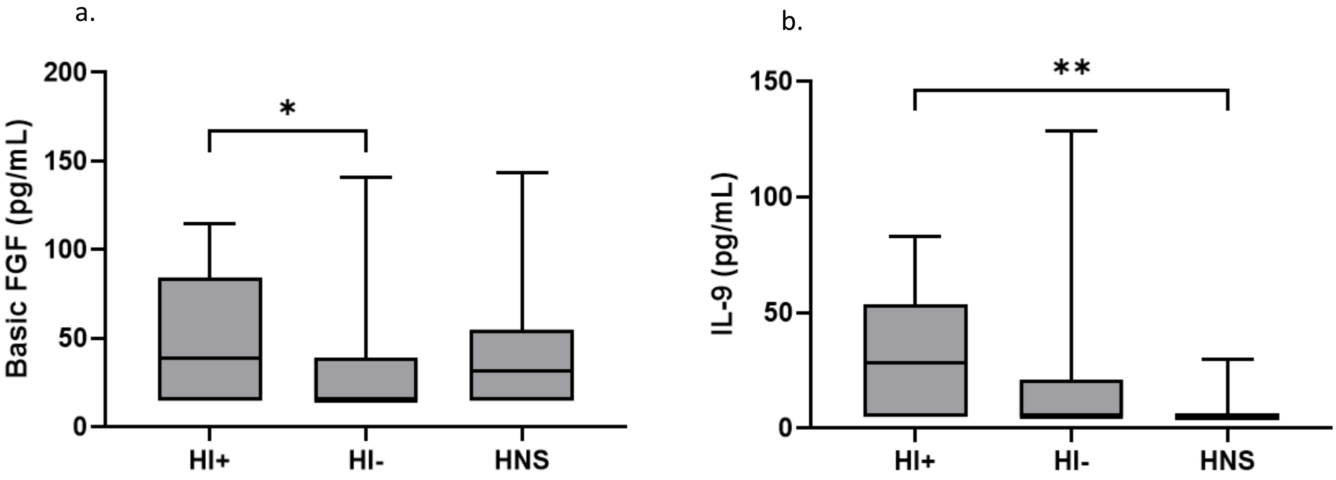

Figure S4: Unvalidated Measurements of Sputum Biomarkers in COPD HI$^{+ve,}$ COPD HI^{-ve} and HNS: (a) IL-17A, data is presented as individual concentrations and the median concentration, data analysed using Krushkal-Wallis test with Dunns post hoc test. (b) IP-10, data is presented as individual concentrations and the median concentration. (c) TNF-a, data is presented as individual concentrations and the median concentration, data analysed using Krushkal –Wallis test with Dunns post hoc test.

Tables:

Table S1: Assay Kits

Analyte	Kit	Manufacturer	Manufacturer reported assay range
MPO	Quantikine ELISA Kit, Catalogue No. DMYE00B	R&D Systems	0.2 -10 ng/mL
IL-8	Human IL8/CXCL8 Quantikine ELISA Kit, Catalogue No. D8000C	R&D Systems	31.2 – 2000 pg/mL
IL-1β<br>IL-6<br>TNF-α	Milliplex MAP Human High Sensitivity T Cell Panel, Catalogue No. HSTCMAG-28SK.	Merck Millipore	0.49 – 2000 pg/mL<br>0.18 – 750 pg/mL<br>0.45 – 1750 pg/mL
Basic FGF<br>Eotaxin	Bio-Plex Pro Human Cytokine 27-plex	Bio-Rad	3.26 – 3341 pg/mL<br>0.14 – 2281 pg/mL

G-CSF	Assay, Catalogue No.	6.35 – 104106 pg/mL
GM-CSF	M500KCAF0Y	0.48 – 7846 pg/mL
IFN-y		1.57 – 25665 pg/mL
IL-1β		0.29 – 4672 pg/mL
IL-1RA		6.21 – 34949 pg/mL
IL-2		1.29 – 21178 pg/mL
IL-4		0.19 – 3064 pg/mL
IL-5		3.63 – 59499 pg/mL
IL-6		0.38 – 6244 pg/mL
IL-7		1.92 – 31475 pg/mL
IL-8		0.85 – 13992 pg/mL
IL-9		3.62 – 31527 pg/mL
IL-10		1.06 – 17427 pg/mL
IL-12p70		1.43 – 23425 pg/mL
IL-13		0.31 – 5157 pg/mL
IL-15		12.42 – 203426 pg/mL
IL-17A		2.44 – 39972 pg/mL
IP-10		3.41 – 34953 pg/mL
MCP-1		0.53 – 8755 pg/mL
MIP-1a		0.12 – 1218 pg/mL
MIP-1b		1.41 – 1439 pg/mL
PDGF-BB		7.12 – 37133 pg/mL
RANTES		16.72 – 26467 pg/mL
TNF-a		3.33 – 54566 pg/mL
VEGF		18.01 – 149830 pg/mL

Table S2: Selection of Assay MRD from Matrix Dilution Assessment

**Subject #20811 Raw Data**					
**Replicate**	*Sample Dilution*				
	**Neat**	**1:2**	**1:4**	**1:8**	**1:16**
1	15.942	5.856	8.455	6.571	3.644
2	16.394	6.228	8.255	6.257	3.702
Mean (pg/mL)	16.168	6.042	8.355	6.414	3.673
Standard Deviation	0.320	0.263	0.141	0.222	0.041
CV (%)	1.977	4.354	1.693	3.462	1.117

**Subject #20811 Matrix Dilution Assessment**			
Sample *Proposed MRD: Neat*	Uncorrected Observed Mean Concentration	Expected Concentration	Difference (%)
Neat	16.168	N/A	N/A
1:2	6.042	8.084	74.740
1:4	8.355	4.042	206.705*
1:8	6.414	2.021	317.368*
1:16	3.673	1.011	363.483*
Sample *Proposed MRD: 1:2*	Uncorrected Observed Mean Concentration	Expected Concentration	Difference (%)
1:2	6.042	N/A	N/A

1:4	8.355	3.021	276.564*
1:8	6.414	1.511	424.628*
1:16	3.673	0.755	486.329*
Sample *Proposed MRD: 1:4*	Uncorrected Observed Mean Concentration	Expected Concentration	Difference (%)
1:4	8.355	N/A	N/A
1:8	6.414	4.178	153.537*
1:16	3.673	2.089	175.847*
Sample *Proposed MRD: 1:8*	Uncorrected Observed Mean Concentration	Expected Concentration	Difference (%)
1:8	6.414	N/A	N/A
1:16	3.673	3.207	114.531

Subject #4008 Raw Data

**Replicate**	*Sample Dilution*				
	**1:4**	**1:8**	**1:16**	**1:32**	**1:64**
1	4.863	2.382	1.298	0.554	0.261
2	4.760	2.610	1.348	0.652	0.285
Mean (pg/mL)	4.812	2.496	1.323	0.603	0.273
Standard Deviation	0.073	0.161	0.035	0.069	0.017
CV (%)	1.514	6.459	2.672	11.492	6.216

Subject #4008 Matrix Dilution Assessment

Sample *Proposed MRD: 1:8*	Uncorrected Observed Mean Concentration	Expected Concentration	Difference (%)
1:8	2.496	N/A	N/A
1:16	1.323	1.248	106.010
1:32	0.603	0.624	96.635
1:64	0.273	0.312	87.500

*Value outside of acceptance criteria.

Table S3: MPO, IL-8 and 3-Plex Luminex Assay Validated Quantitative Ranges

**Analyte**	**Quantitative Range**
MPO	0.125 – 10 ng/mL
IL-8	34.215 – 2000 pg/mL
IL-1β	0.890 – 2000 pg/mL
IL-6	0.479 – 750 pg/mL
TNF-α	0.967 – 1750 pg/mL

Table S4: 27-Plex Luminex Assay Validated Quantitative Ranges

**Analyte**	**Quantitative Range (pg/mL)**
Basic FGF	0.920 – 3747.81
Eotaxin	0.090 – 372
G-CSF	17.370 – 17790.50

GM-CSF	0.440 – 1816.25
IFN-y	2.40 – 2455
IL-1β	0.270 – 1116.75
IL-1RA	10.76 – 11022.69
IL-2	0.420 – 6952.75
IL-4	0.210 – 855.50
IL-5	5.340 – 21872.75
IL-6	0.420 – 1729.50
IL-7	2.610 – 2667.50
IL-8	0.810 – 3314.75
IL-9	1.230 – 5031.25
IL-10	0.810 – 3321
IL-12p70	0.470 – 7666.75
IL-13	0.09 – 1399.75
IL-15	18.020 – 73804
IL-17A	2.670 – 10950.50
IP-10	5.650 – 5789.75
MCP-1	0.580 – 598.06
MIP-1a	0.360 – 365.25
MIP-1b	1.440 – 1476.50
PDGF-BB	39.400 – 2521.81
RANTES	5.460 – 5594.75
TNF-a	13.410 – 13791.25
VEGF	16.900 – 4325.75

Table S5: Matrix Dilution Precision Data

Analyte	Average %CV
MPO	1.22
IL-8	5.27
IL-1β	11.23
IL-6	7.79
TNF-α	8.88
Basic FGF	2.16
Eotaxin	4.33
G-CSF	7.34
GM-CSF	9.29
IFN-y	5.35
IL-1β	3.33
IL-1RA	3.02
IL-2	13.38
IL-4	12.75
IL-5	16.19
IL-6	6.18
IL-7	7.89
IL-8	2.83
IL-9	6.12
IL-10	<LLOQ
IL-12p70	15.74
IL-13	<LLOQ
IL-15	8.56

IL-17A	7.02
IP-10	3.03
MCP-1	3.98
MIP-1a	3.63
MIP-1b	3.92
PDGF-BB	3.81
RANTES	3.68
TNF-a	7.10
VEGF	13.47

Matrix dilution precision data is presented as the average %CV derived from samples analysed in duplicate at varying dilutions, with the MRD acting as the reference matrix.

Table S6: Establishment of Calibration Curve Precision Data

Analyte	Average %CV
MPO	3.82
IL-8	5.21
IL-1β	6.89
IL-6	7.04
TNF-α	4.82
Basic FGF	8.26
Eotaxin	5.04
G-CSF	9.08
GM-CSF	9.50
IFN-y	5.70
IL-1β	7.98
IL-1RA	8.52
IL-2	4.70
IL-4	9.62
IL-5	5.42
IL-6	13.22
IL-7	7.17
IL-8	4.97
IL-9	6.84
IL-10	7.70
IL-12p70	5.65
IL-13	11.82
IL-15	5.73
IL-17A	3.94
IP-10	9.07
MCP-1	4.40
MIP-1a	8.66
MIP-1b	5.21
PDGF-BB	<6 calibrators
RANTES	6.15
TNF-a	4.65
VEGF	<6 calibrators

Data is presented as the average %CV of validated standard calibrators from data collated from 3 independent runs.

Table S7: 27-Plex Luminex Assay Analyte Specific Standard Calibrators

Analyte	No. Of Validated Standard Calibrators
Basic FGF	7
Eotaxin	7
G-CSF	6
GM-CSF	7
IFN-y	6
IL-1β	8
IL-1RA	6
IL-2	7
IL-4	7
IL-5	7
IL-6	7
IL-7	7
IL-8	6
IL-9	7
IL-10	7
IL-12p70	8
IL-13	8
IL-15	7
IL-17A	7
IP-10	6
MCP-1	6
MIP-1a	7
MIP-1b	7
PDGF-BB	<6
RANTES	6
TNF-a	7
VEGF	<6

Table S8: Lower Limit of Quantification and Limit of Detection

Analyte	LLOQ	LLOQ Inter-Assay Precision (%CV)	LOD
MPO	0.13	10.15	0.00
IL-8	34.22	8.08	1.86
IL-1β	0.89	10.15	0.24
IL-6	0.48	15.93	0.22
TNF-α	0.97	8.92	0.27
Basic FGF	3.53	14.36	1.08
Eotaxin	0.07	3.27	0.06
G-CSF	28.40	17.86	16.89
GM-CSF	0.46	23.59	0.12

IFN-y	3.39	9.93	1.18
IL-1β	0.06	24.98	0.05
IL-1RA	5.01	17.04	0.11
IL-2	1.60	5.02	0.36
IL-4	0.16	18.20	0.05
IL-5	1.11	18.51	0.00
IL-6	0.35	6.59	0.09
IL-7	2.05	11.27	1.38
IL-8	2.51	10.18	0.21
IL-9	2.49	7.13	0.00
IL-10	1.22	13.60	0.29
IL-12p70	0.39	19.74	0.08
IL-13	0.09	7.76	0.09
IL-15	15.37	9.50	3.08
IL-17A	2.27	4.85	0.28
IP-10	5.57	20.88	2.75
MCP-1	0.52	12.06	0.20
MIP-1a	0.08	16.82	0.06
MIP-1b	0.24	11.86	0.07
PDGF-BB		Unvalidated Assay	
RANTES	3.99	10.90	0.70
TNF-a	2.70	1.07	0.72
VEGF		Unvalidated Assay	

For MPO, IL-8 and the 3-Plex Luminex assay, LLOQ precision data is presented as the average CV of 6 LLOQ replicates per run, across 3 individual runs. For the 27-Plex assay, LLOQ precision data is presented as the CV of 2 replicates of the lowest validated standard calibrator per run, across 3 individual runs. MPO data is presented as ng/mL and all other analytes as pg/mL.

Table S9: Cohort B: Unvalidated Measurements of Sputum Biomarkers

**Analyte**	**COPD (n = 81)**	**HS (n = 15)**	*p* Value (COPD vs HS)	**HNS (n = 26)**	*p* Value (COPD vs HNS)
IL-7 (pg/mL)	29.76 [10.44-137.80]	10.44 [10.44-91.25]	0.98	19.15 [10.44-78.44]	0.65
IL-9 (pg/mL)	4.92 [4.92-128.70]	4.92 [4.92-33.96]	>0.99	4.92 [4.92-29.94]	0.02
Basic FGF (pg/mL)	14.64 [4.64-140.70]	14.64 [14.64-125.40]	>0.99	31.24 [14.64-143.10]	>0.99

Data presented as median [range] as appropriate. HS and HNS compared to COPD using Kruskal-Wallis with Dunns post-hoc analysis. IL-12p70, GM-CSF, PDGF-BB and VEGF were not detectable.

Table S10: Cohort B: Sputum Neutrophil Percentage and Unvalidated Supernatant Cytokine Correlations in COPD, HS and HNS

Analyte	COPD (n = 79)	HS (n = 14)	HNS (n = 25)
IL-7 (pg/mL)	rho = 0.1692 p=0.14	rho = 0.1393 p=0.63	rho = -0.2183 p=0.28
IL-9 (pg/mL)	rho=0.2585 p=0.03	rho=0.6761 p=0.20	rho=0.0000 p>0.99
Basic FGF (pg/mL)	rho=0.4231 p=0.0001	rho=0.4380 p=0.12	rho=0.0697 p=0.74

Data is presented as rho and p values. Results analysed using Pearson's coefficient correlation test for parametric data and Spearman's rank test for non-parametric data.

Table S11: Cohort A Inhaled Medications

Inhalor Combination	COPD (n=30)
ICS Use (%)	86.7
LABA+LAMA+ICS (%)	76.7
LABA + ICS (%)	6.7
LAMA + ICS (%)	3.3
LABA+LAMA (%)	3.3
ICS Only (%)	0.0
LABA Only (%)	3.3
LAMA Only (%)	6.7
No inhaled medication (%)	0.0

Table S12: Cohort B Inhaled Medications

Inhalor Combination	COPD (n=81)
ICS Use (%)	69.1
LABA+LAMA+ICS (%)	54.3
LABA + ICS (%)	11.1
LAMA + ICS (%)	2.5
LABA+LAMA (%)	11.1
ICS Only (%)	1.2
LABA Only (%)	0.0
LAMA Only (%)	11.1
No inhaled medication (%)	4.9

Table S13: Cohort A: Paired Stable vs Exacerbation Sputum Cell Counts

Characteristic	Stable	E0	*p* Value
Sputum Neutrophil (%)	82.75 [48.00-97.50]	92.00 [67.00-98.10]	0.0161
Sputum Macrophage (%)	11.88 [1.50-31.50]	6.75 [1.70-50.00]	NS
Sputum Eosinophil (%)	0.88 [0.00-7.00]	0.25 [0.00-3.75]	NS
Sputum Lymphocyte (%)	0 [0-1.5]	0 [0-0]	NS
Sputum Epithlial (%)	2.875 [0-14]	1 [0-5.5]	0.0038

NS: Non-significant

Table S14: Cohort B: ICS Effect on Sputum Cytokine Expression

Analyte	COPD ICS	COPD Non-ICS	*p* Value
IL-1β	8.93 [0.28-608.60]	7.61 [0.28-947.60]	0.89
IL-1RA	6947 [2198-25584]	7052 [2254.31785]	0.57
IL-2	6.80 [6.80-28.97]	6.80 [6.80-6.80]	0.18
IL-4	5.00 [0.84-28.73]	5.08 [0.84-15.22]	0.90
IL-6	86.29 [13.71-485.10]	77.51 [16.78-296.00]	0.88
IL-8	2954 [491.70-25879]	2526 [354.9-26518]	0.98
IL-17A	10.68 [3.24-58.92]	10.68 [3.24-34.04]	0.51
Eotaxin	55.25 [4.73-253.30]	56.49 [4.96-94.41]	0.40
G-CSF	532.30 [69.48-3710]	497.30 [69.48-2784]	0.79
IFN-y	56.84 [9.60-165.10]	49.25 [9.60-256.60]	0.99
IP-10	6405 [587.9-46318]	8436 [1101-46318]	0.68
MCP-1	81.86 [13.69-1946]	118.60 [14.85-1071]	0.16
MIP-1a	19.41 [0.36-304.00]	27.69 [1.00-147.40]	0.94
MIP-1b	114.30 [1.44-2417]	171.30 [5.61-751.10]	0.69
TNF-a	56.89 [13.40-5561]	66.50 [13.40-877.00]	0.95

Data presented as median [range] as appropriate. Comparisons analysed using Mann Whitney tests.

<u>References:</u>

1. Garcha, D.S., et al., *Changes in prevalence and load of airway bacteria using quantitative PCR in stable and exacerbated COPD.* Thorax, 2012. **67**(12): p. 1075-80.
